# At-Home Pupillometry using Smartphone Facial Identification Cameras

**DOI:** 10.1145/3491102.3502493

**Published:** 2022-04-29

**Authors:** Colin Barry, Jessica De Souza, Yinan Xuan, Jason Holden, Eric Granholm, Edward Jay Wang

**Affiliations:** Department of Electrical and Computer Engineering, University of California: San Diego La Jolla, California, USA; Department of Electrical and Computer Engineering, University of California: San Diego La Jolla, California, USA; Department of Electrical and Computer Engineering, University of California: San Diego La Jolla, California, USA; Center for Mental Health Technology, University of California: San Diego La Jolla, California, USA; Center for Mental Health Technology, University of California: San Diego La Jolla, California, USA; Department of Electrical and Computer Engineering, University of California: San Diego La Jolla, California, USA

## Abstract

With recent developments in medical and psychiatric research surrounding pupillary response, cheap and accessible pupillometers could enable medical benefits from early neurological disease detection to measurements of cognitive load. In this paper, we introduce a novel smartphone-based pupillometer to allow for future development in clinical research surrounding at-home pupil measurements. Our solution utilizes a NIR front-facing camera for facial recognition paired with the RGB selfie camera to perform tracking of absolute pupil dilation with sub-millimeter accuracy. In comparison to a gold standard pupillometer during a pupillary light reflex test, the smartphone-based system achieves a median MAE of 0.27mm for absolute pupil dilation tracking and a median error of 3.52% for pupil dilation change tracking. Additionally, we remotely deployed the system to older adults as part of a usability study that demonstrates promise for future smartphone deployments to remotely collect data in older, inexperienced adult users operating the system themselves.

## 1 INTRODUCTION

Pupillometry is enabled by specialized devices called pupillometers that measure the pupil. The pupil is the part of the eye inside the colored iris that allows light to enter into the retina, where light-sensitive cells react to the incoming light that leads to our visual experience [16]. Like an aperture of a camera, the pupil can dilate/shrink to increase/decrease the amount of light that enters the eye or adjust to the depth of the field of view [16]. However, the pupil does not only respond to light. The pupil is connected with a number of complex neurological processes, leading to pupillary response having a correlation with cognition, arousal, and emotion [16][29]. This connection between pupillary response and neurological processes has led to clinical research around how pupillary response is connected to neurological diseases like Alzheimer’s disease, schizophrenia, and Parkinson’s Disease [17] [6] [13] [10] [24] [25]. Clinical researchers and medical experts have thus speculated on the possible functionality of mobile pupillometers in telemedicine ranging from diabetes management [4] to Alzheimer’s screening [6].

From Alzheimer’s and Parkinson’s Disease screening in older adults to ADHD detection in children, previous research indicates that pupillometry has the potential to make a significant impact on the screening and care for a number of neurological diseases [6] [8] [17] [6] [13] [10]. Unfortunately, clinical grade pupillometers that work on a wide variety of eye colors and at an approachable cost are not widely available and much of the findings cannot be translated towards end-consumer and at-home patient use.

Expanding the research and applications surrounding pupillary response hinges on patient access to pupillometers. This has been done to some extent with eye-tracking research enabled by RGB cameras on mobile devices. However, a key consideration for medical and research grade pupillometers is the difficulty of accurate pupil measurement. Given that the pupil is responsive to light, emotion, and even respiratory fluctuations, pupil-based biomarkers often require a high accuracy measurement[20] [16] [30]. Thus, high accuracy measurements are key to a successful deployment of pupillary response research and applications. These expensive medical devices generally contain one or more near-infrared (NIR) cameras that easily discern the pupil from the iris. In the NIR spectrum, the pupil has a significantly different refection than the pupil and the rest of the eye [12]. Given that pupillometers often record pupil response while a participant performs a test, the pupillometer cameras generally have both high resolution and a high frame rate.

In our work, we leverage the hardware on emerging smartphones equip with facial recognition to demonstrate the novel potential of these smartphones for delivering clinical grade pupillometry to the masses. To our knowledge, we are the first to create and validate a smartphone pupillometer with absolute measurements. In addition to demonstrating the functionality of the smartphone device, we test the usability of our system during remote deployments to older adults. Older adults are one of the target population for much of the neurological research linking pupillary reflex to neural degenerative diseases like Alzheimer’s Disease and Parkinson’s Disease. As such it is paramount to ensure that the cheap, smartphone-enabled pupillometers can be easily operated by older adults with minimal intervention. In this paper, we present the usability findings in our development and testing of our smartphone system and user interface built for older adults with little to no previous experience using smartphones or other touch screen devices.

Smartphone-based pupil measurements will expand research out of the lab into the homes of patients to enable accurate measurements with higher frequency in a comfortable environment. Also, this research facilitate investigations into the effects of diuretic effects, life stresses, and environmental factors that may affect pupil-based biomarkers.

### 1.1 Limitations of Previous Work

#### 1.1.1 Absolute Size.

Note that some previous work has reported their findings with absolute errors in millimeters, however each of these works specifically acknowledges that their system does not actually measure millimeter change in absolute [15][19]. Instead, previous systems measure percent change in pixels, which is converted to millimeters based on a single scaling constant regardless of distance of the eye to the camera [15], or by scaling measurements based on the values in the ground truth measurement [11]. The use of millimeters in reporting by these prior work helps to bring physical meaning to the reported performance, however, it should be noted that they do not have a ground truth distance measurement and thus do not actually measure absolute size of the pupil in millimeters. The findings in these work can only support use cases around tracking dilation change due to major stimuli (relative measure) and cannot support use cases that require a measure of absolute pupil size (absolute measure) which may be used for wakefulness or cognitive load.

#### 1.1.2 Light.

To the best of our knowledge, the systems in prior work that leverages RGB cameras require the smartphone flash to also be on and directed towards the user’s eye to help with illumination of the eye. This introduces significant limitations for the types of tests that can be performed. Even for the light reflex tests performed in each study, the smartphone systems can only record the portion of the test where the light is on, when in general, a light reflex test involves turning the light on and of during the measurement to capture both the constriction and recovery of the pupil [25]. Furthermore, in tests that elicit smaller dilatory effects, such as cognitive battery tests like the digit span, a strong light stimulus may override the effect.

#### 1.1.3 Validation.

In some previous work, the smartphone accuracy is demonstrated during a pupillary light reflex test [15] [11]. While this is a test commonly used for a variety of purposes and it may have been necessary given the lighting requirements of previous systems, only testing the system in this condition can result in exaggerated results. The pupillary light reflex test generally elicits a very large change in pupil dilation. Since the results are calculated in some form of percent change, the large range of the pupillary light reflex can lead to low error in percent change despite a much larger absolute error.

#### 1.1.4 RGB Cameras.

All previous work relies on RGB smartphone cameras [15] [11] [19]. These works report difficulties and differences in results based on eye colors because the iris and the pupil appear very close in color in RGB cameras, especially for darker iris colors. Neice et al. [19] state in the 23 percent of their measurement scans which failed to detect the pupil or iris, the strong majority of the scans were from darker eye colors. Newer smartphones are coming equipped with NIR cameras for facial recognition for bio-metric security. In this work, we investigate the potential to use these low resolution and frame-rate NIR cameras for pupillometry.

### 1.2 Contribution

To enable highly accurate smartphone-based pupillometry, our major contributions of this work are as follows:

- Development and validation of an accurate pupillometer, enabled by smartphone facial recognition camera systems, that measures both relative pupil size change and absolute pupil measurements in millimeter.
- Usability findings on the feasibility of deploying smartphone pupillometers to older adults to remotely conduct pupil response tests.

## 2 RELATED WORK

### 2.1 Pupillometry Applications

There is a significant research foundation on the applications of pupillometry towards a variety of neurological diseases and use cases. There is a long history of using pupil response for a variety of screening and insights into neurological processes [29]. More recent research demonstrates that pupil response is linked to more than cognition, arousal, or emotional state, and suggests the possibility that the early damage of neurodegenerative diseases may also be detectable from pupillary response tests [7] [3] [6].

### 2.2 Mobile Pupillometry

#### 2.2.1 Eye-tracking vs. Pupillometry.

The main body of work regarding eye-related sensing explores eye-tracking or gaze estimation rather than pupillary response [9][27][28]. There are major differences between eye-tracking and pupillometry in mobile device measurements. For eye-tracking, the measurement must estimate the rotation of the eyeball based on the pupil and iris location in the image. This measurement is generally an angle, so absolute size of the pupil/eyeball is not important. Also, the pupil and iris move together so accurate discrimination of the pupil from the iris is less important. In pupillometry, pupil size estimation is the goal, so discerning the iris and pupil is essential. This is especially significant in people with darker irises because the iris and the pupil are difficult to discern in the visible light spectrum (both often appear black in RGB images).

Outside of the smartphone domain, there exist a number of other devices for pupillometry research that report pupil diameter or radius. These include the Tobii eye tracker [22] and Pupillabs glasses [21], which are commonly used in pupil research. Both of these devices are more geared towards gaze tracking rather than absolute pupil size estimation. Although both devices report pupil size, the devices do not have any accuracy validations for their pupil size estimation. In addition, they are not cheap or accessible like smartphones. With these limitations, these solutions are poor options for expanding pupillometry beyond laboratory studies towards end-users and at-home research.

#### 2.2.2 Smartphone Pupillometry.

The most similar application to our work is PupilScreen, a smartphone-based application developed to enable concussion diagnosis in the wild. This application requires a large phone attachment that will allow the user to perform a concussion test [15]. There are some other similar works, but all leverage a smartphone RGB camera for pupil measurements with a specific applications in mind [11]. A recent feasibility study of smartphone pupillometers based on RGB cameras demonstrated that the smartphone pupillometers were unable to correctly detect the pupil or iris in nearly a quarter of the scans [19]. Note that although some previous work reports findings in millimeters, we were not able to find any previous work that calculates and validates absolute measurements against a clinical pupillometer. PupilScreen reports their findings in millimeter scale, however, as noted by the authors, this is calculated by an empirically determined fixed conversion of pixel to millimeter regardless of eye socket depth. This work also based it’s validation against human performance of image labeling instead of against a clinical pupillometer, thus only validating the computer vision algorithm against human labels rather than an absolute measurement by a clinical reference.

Further, prior work has only focused on the use of RGB cameras in smartphones and have not explored the possibility of using the facial recognition cameras on smartphones to perform this task. As discussed above, clinical pupillometers use NIR cameras because of the difference in NIR reflections between the pupil and the iris. An RGB camera have lower contrasts in different lighting conditions, especially for individuals with darker eyes [19] [12]. By repurposing the NIR camera in newer smartphones, we demonstrate that we can achieve higher accuracy within at home environments with minimal constraints or specified lighting conditions. To the best of our knowledge, our work is the first demonstration of pupillometry using the smartphone facial-recognition camera with validated, absolute value measurements against a clinical pupillometer.

## 3 METHODS

We present a novel smartphone pupillometer solution that leverages the NIR camera in smartphone facial recognition systems to measure the pupil. The smartphone pupillometer functions as a 3rd party app and can provide both relative change and absolute pupil measurements without an attachment, add-on, or supplemental hardware. This is achieved by using the secondary front-facing RGB camera to capture a stereovision distance measure between the phone and the eye, then convert a pixel measurement to a millimeter measurement of the pupil diameter.

### 3.1 Repurposing Facial Recognition Technology

The possibility of high accuracy, absolute pupil measurements using a smartphone stems from the inclusion of NIR cameras in newer smartphones for facial recognition purposes. The NIR camera is essential to any robust, high performance pupillometry system because the difference in refection between the pupil and the iris in the NIR spectrum is much more significant than that of the visible spectrum recorded with an RGB camera. We can use the front facing RGB “selfie” camera in conjunction with the NIR camera to obtain stereovision distance measurements needed for capturing absolute distance for converting pixels to mm. For this paper, the system is designed using Android Studio to programmatically access the Google Pixel 4 front facing NIR and RGB cameras.

### 3.2 Distance Measurement and Size Estimation

Given that both camera locations are fixed in the same plane, one can calculate distance from the camera to an point of interest (center of the pupil) based on the disparity between location of the specified point in the two images. First, images from the RGB and NIR cameras are analyzed to return the center of the pupil in both images. Then the relative disparity of the pupil center location is calculated between the two images as shown in the equation below, where x* refers to the horizontal location of the center of the pupil in the image frame.


(1) $$\text{Disparity}=\left|\frac{x_{RGB}^{*}}{\text{RGB}\;\text{Image}\;\text{Length}}-\frac{x_{IR}^{*}}{\text{NIR}\;\text{Image}\;\text{Length}\;}\right|$$

From the disparity calculation, we use an experimentally determined conversion variable (K) to convert the disparity to the distance between the phone and the center of the pupil following equation 2 below. Conventional calculations generally calculate K as a function of the camera lens focal length and the distance between the two cameras, but these values were unavailable to us [18] [14].


(2) $$\text{Distance}=\frac{K}{\text{Disparity}}$$

Once we find the distance between the camera and the image, we estimate the absolute size of the full image using the camera angle of view (*θ*). From the absolute size of the full image, we calculate the absolute size of each pixel. The absolute pixel size is used to determine the absolute size of the pupil diameter, from the decimal number of pixels output from the neural network. This process allows for pupil size estimations beyond the limits of the camera resolution because the diameter estimation can include fractions of a pixel.


(3) $$\text{Pupil}\;\text{Diameter}=2\times \text{radius}\times \frac{2\times \text{Distance}\times \theta }{\text{number}\;\text{of}\;\text{horizontal}\;\text{pixels}}$$

### 3.3 Smartphone Application

We developed an Android application to perform pupil measurements on the Google Pixel 4 using the Camera 2 API. The Google Pixel 4 smartphone is used for all studies and experiments presented in this paper. We developed 2 variations of our application: one for the validation study and one for the deployment study. The 2 variations differ almost exclusively on user interface and are outlined in the Study section.

#### 3.3.1 Data Acquisition.

In both versions of the pupillometer application, pupil response tests are triggered by pressing a button. After the button press, the smartphone verbally instructs the user to “hold the phone over your right eye and cover your left eye with your hand or eye patch.” After the user is given 5 seconds to hold the phone over their eye, the smartphone will initiate the recording and begin verbal instructions for the pupil response test. Due to the limitations of simultaneous camera use, the application takes a RGB picture at the start of the test, then records from the NIR camera for the length of the test, and finally takes another RGB picture at the end of the test.

It is important to note that although this method relies on an RGB image of the pupil to determine distance, it does not require pupil estimation on the RGB image. As discussed above, discerning the pupil from the iris is difficult in RGB images of darker eyes. In our method, the system must only find the center of the pupil in the RGB image, which can be accurately estimated as the center of the iris. Discerning the pupil from the iris in the RGB image is not necessary. The diameter estimation of the pupil is calculated from only the NIR image frames.

### 3.4 Data Processing

The data processing is performed of device after data acquisition using python. First, the data is preprocessed using image rotations, gray scaling, histogram equalization, and median blur. The high performance neural network proposed by Eivazi et. al. [5] is used to locate the pupil center and diameter of each image frame for both the NIR and RGB cameras. This model is chosen as a validated process with high performance across pupil datasets that can be implemented in real-time. From the digital images, we use stereovision calculations from the RGB and NIR images to provide a pupil size estimate in millimeters rather than pixels. The baseline distance measurement is determined with the first RGB picture and the first NIR image frame after auto focus and white balance NIR camera operations are completed. If there is a blink or other issue detected with the first RGB image and IR frame pair then the second RGB image and last IR frame pair is used instead. The pupil size measurement uses the distance from the baseline measurement for all frames of the NIR video feed.

### 3.5 Distance Validation

To test the distance and size estimation, we conducted a short preliminary bench-top experiment. The experiment is setup using a 3D printed stair structure to lift the phone off of the table in 0.5cm increments between 2–4cm and 1cm increments from 4–7cm. We estimate that 2–7cm is about how far the phone could be held up to the eye in our application. A piece of paper with a 1cm circle printed in black ink (shown in blue on Fig 3) is situated under the camera. The measurement is performed 5 times at each step by removing and replacing the phone each time.

Based on the results from figure 3, the smartphone should be positioned within 4cm of the pupil during testing to ensure quality distance estimates. We note that at farther distances, the estimation becomes less accurate as the disparity is no longer big enough for the resolution to differentiate.

## 4 STUDY SECTION

The following section details the procedure of two studies. First is a validation study around the performance of the smartphone pupillometer compared to a gold standard clinical pupillometer. Second is a usability study where the smartphone pupillometer was deployed to 15 older adult participants undergoing cognitive assessments.

### 4.1 Study 1: Validation Against a Clinical Pupillometer

To validate the accuracy of the smartphone pupillometer, we compare our system to the Neuroptics PLR 2000 pupillometer, a gold standard for pupil measurements in research and clinical studies. [23]. We perform validations with both a light reflex test and a digit span recall test. The light reflex test allows comparisons to previous work and provides measurements across a large range of pupil sizes. On the other hand, the digit span recall test challenges the smartphone-based system with a significantly lower signal-to-noise ratio. To demonstrate the robustness of the system, the tests were performed on a variety of people in the laboratory and home locations with no specified restrictions or lighting qualifications. The study is meant to simulate normal living conditions that could be reasonably recreated by most individuals. As such, the tests were conducted during business hours in common spaces (open rooms in the laboratory or in homes) with the lights on. We did not perform tests in worst-case scenario conditions such as absolute darkness, maximal light, outdoor environments, etc. As shown in Table 1, we tested on a range of people with varying ethnicity and eye colors to do our best to ensure our system functioned equitably. The most important of these scenarios are the participants with darker eyes because the pupil is less discernible from the dark iris in the visible light spectrum. As such, the majority of our test subjects have darker iris colors to emphasize the performance on cases where RGB systems have the most difficulty [19].

**Setup** A participant’s eyes are simultaneously recorded with the Neuroptics PLR 2000 and the smartphone. The participant’s right eye is recorded with the Nueroptics PLR 2000 while the participant’s left eye is recorded with the smartphone. This is permitted because of the tight coupling of pupil dilations between the two eyes, referred to as consensual pupil response [26]. Finally, the measurement from the Neuroptics PLR 2000 and the smartphone videos were manually aligned.

The participant holds the smartphone without assistance to capture the recording. The smartphone contains no add-ons or attachments for the recording session. With both devices simultaneously recording, the smartphone first administers a light reflex test then administers a digit span recall test.

**Analysis** Two analysis are performed for comparing the smartphone’s performance against the clinical pupillometer. First is a comparison of the absolute pupil size based on the stereovision distance measurement. Second is a comparison of relative pupil size. This comparison validates that the smartphone pupillometer accurately follows the changes in pupil size and lends a metric that can be more easily evaluated against other mobile pupillometry studies. We calculate relative pupil size using a mean normalization.

#### 4.1.1 Validation Study Application.

Beyond collecting data, the main purpose of the user interface of the validation study is to enable the researcher to monitor the data acquisition for data quality. As such, the validation study application is meant to be used in parallel with Vysor, software that allows desktop viewing and control of a smartphone. In the application, the user is first prompted to input their ID number, then they proceed to the testing screen. On the testing screen, a button click will trigger the phone to start a pupil response test.

#### 4.1.2 Test 1: Pupillary Light Reflex Test.

The light reflex test demonstrates the accuracy of our system across a wide range of pupil dilation measurements, represents a common pupil response test performed in research, and mimics the types of tests done to validate previous smartphone-based systems. In this test, the phone screen brightness changes to elicit a pupillary response. During the test, the screen brightness starts at the minimum brightness, then turns to the maximum brightness setting for about 5 seconds before returning to the minimum brightness for approximately 3 more seconds. The changes in brightness are enacted automatically with no manual intervention. Figure 5 shows an example measurement of the expected pupillary response of pupil dilation during increased brightness and pupil constriction during changes from a darker screen (minimum brightness).

#### 4.1.3 Test 2: Digit Span Recall Test.

The digit span recall test is a memory based test that elicits pupil response based on cognitive load. The pupil dilation is correlated with cognitive load such that increasing cognitive load increases pupil size. Prior work by Granholm et al. [6] suggests that pupillary response from the digit span recall test may provide insight into early detection of mild cognitive impairment in Alzheimer’s Disease. In a digit span recall test, a subject is asked to remember increasingly longer spans of number sequences. Often, the user is asked to remember spans of length 3, 4, 5, 6, 7, 8, and 9 number of digits. At each digit span length, there are 3 trials for a total of 25 trials. The expected result of the pupillary response is for the pupil size to increase with difficulty across trials until the participant reaches their memory capacity and psychologically gives up.

For the validation, we chose to record a single digit span recall test trial of 9 digits for each participant to avoid having 25 recordings for each participant. For a single trial, the expected result is for the pupil dilation to increase as more digits are read aloud by the smartphone as shown in Figure 5

### 4.2 Study 2: Usability Study

The purpose of our system is to enable pupillometry beyond lab settings. Based on prior research on the relation between pupillary response and neurological diseases, a large target population for accessible pupillometry is older adults, age 55 and older [6]. As such, we targeted a group of older adults enrolled in an Alzheimer’s cognition study to participate in an at-home usability study using our system.

#### 4.2.1 Smartphone Deployment to Older Adults.

To test our smartphone-based system performance for research style applications, we deployed smartphones to 15 participants within the target population as approved by the Institutional Review Board (Project #210437X). The participants are recruited from previous studies at UCSD with relevant screening for eye conditions and cognitive impairment. Unlike the validation study, the participant’s also received a 3D printed passive scope attachment to aid in usability. The participants did not receive any other equipment (the usability study did not include a direct comparison to a clinical pupillometer). The participants had no prior instruction on how to use the system. The smartphone was dropped of to the participant’s home and the participant received a 20 to 30 minute zoom video call to receive basic instructions on how to use a smartphone and self administer the digit span recall test. On Zoom, the researcher supervising the test has no control over the smartphone. With the instruction from a researcher over Zoom, the participant must turn the phone on and navigate to the testing application. The testing application then instructs the participant on how to self administer the digit span recall test using audio instructions and example images. The participant may ask questions and receive additional instruction on how to self administer tests from the researcher on the Zoom video call. The researchers have no ability to view collected data or control the smartphone until after the phone is returned from a deployment. During the smartphone deployment, qualitative data is collected through interviews conducted at the beginning and end of the deployment as well as observational notes from the zoom video call.

#### 4.2.2 Smartphone Application Functionality.

Based on the NIR pupillometry function described in sections 1 and 2, we introduce a pupillometry app to administer research tests while recording the participant’s pupil. The application gives participants instructions throughout the tests and notifies users when the recordings start and stop to ensure the user is ready for the tests. To help ensure data quality, the smartphone also requires the phone to be positioned in front of the face to record data. The study takes place inside the patients’ own homes under natural settings (ambient light, noise, seating and table setup, etc).

#### 4.2.3 Scope Attachment.

Based on preliminary testing and clinician feedback, we anticipated that older adult users would struggle to properly position and hold the phone to record their pupil by themselves. As such, we provided a 3D printed scope attachment to the smartphone that helps to guide the participant in properly positioning the phone with the pupil in front of the camera during a test. This was important because many participants have never used a smartphone before and may be unaware of where the front-facing smartphone camera is located and how to position the camera in front of the eye. Also, this could help ensure that the participants could more easily hold the phone steadily to reduce shaking or drastic distance changes from the eye.

#### 4.2.4 Deployment Application.

Unlike the validation version of the application, the deployment application focuses on usability for older adults of low technology literacy. In this application, we try to minimize the number and variety of user actions while maximizing the simplicity and enlarging button/text size. The flow of the application is shown below in Figure 2.

## 5 RESULTS

### 5.1 Pupillary Light Reflex Validation Test

In the performance validation test, the pupil size is reported from both the smartphone pupillometer and the clinical pupillometer. The average range across participant’s pupil dilation during a pupillary light reflex test measured by the clinical pupillometer is 2.86mm. Figure 7 shows the individual performance of tracking of the pupil by the smartphone pupillometer, along with the regression and Bland-Altman plot of the absolute and relative comparisons. On average, the smartphone pupillometer has an absolute error of 0.39mm and a relative error of 5.02%. However, the average is heavily influenced by the outlier measurement of participant 4 (P4), with an error of more than 3 standard deviations from the mean and the absolute error. It is difficult to determine the exact cause of the poor measurement for P4, but some of the known issues with the system outlined in section 6.2.1 influenced the measurement. When considering the the median error, the tracking error is 0.27mm and 3.52%. Figure 8 shows examples of the trajectory of pupil dilation tracked over time in different performance quality.

### 5.2 Digit Span Recall Validation Test

In the digit span recall test performance validation, the pupil size is reported from both the smartphone pupillometer and the clinical pupillometer in the same way as the light reflex test. The average range across participant’s pupil dilation during a pupillary light reflex test measured by the clinical pupillometer is 1.41mm. Figure 9 shows the individual performance, along with the regression and Bland-Altman plot of the absolute and relative comparisons. The digit span recall test has higher error than the light reflex test, especially for relative percent change comparisons. On average, the smartphone pupillometer has a mean absolute error of 0.96mm and a relative error of 12.86%. The average median error is 0.62mm or 9.37%. Figure 10 shows the absolute and relative comparisons for different performance levels.

The results from P9 and P11 are excluded due to issues encountered with the ground truth pupillometer measurement. The measurement problems with P9 were caused by user error from the researcher. The smartphone recordings of P11 show the participant blinked more that 15 times in a time span of a few seconds that generated problems for the clinical pupillometer measurement. Note that most of the participants blinked a few times during the recording, but the issue arose because of continuous, rapid blinking.

### 5.3 Usability Study Results

The goal of the usability study is to explore the effectiveness of remotely deploying smartphones to older adults who may not have experience with smartphones or touch screen devices. To quantify the results to some extent, we qualify collected pupil data as “usable” or “unusable” based on how easy it would be to analyze the data without errors. We define “usable” here to signify data where the pupil is clearly visible and unobstructed in frame as exemplified in Figure 11. If parts of the pupil are obstructed in anyway or the pupil is partially out of frame the data is considered unusable. In these definitions, we aim to bias towards usable data being considered unusable so that we can develop the best possible methods for achieving high quality data in future deployments.

All participants positioned the phone at the eye and initiated the tests successfully. Of the 15 total participants, 8 patients had data at least 80 percent usable. 2 participants had data at least 50 percent usable. Lastly, 5 participants had poor data quality that was mostly unusable.

In the example images from the deployment are shown in Figure 11, examples b and c demonstrate the main issue of eyelid and eyelash obstruction found in the deployment data. We did not experience this problem in any of our preliminary work or in any of the validation studies likely because our previous work focused on a younger population. The issue of drooping eyelids/eyelashes occluding the view of the pupil is the most significant problem because it persists throughout the data measurements. The eyelid will cover parts of or the entirety of the eye, whereas eyelashes will make the pupil appear blurry as it casts shadows and reflect the IR light.

In addition to the major issue of low hanging eyelids, a few other problems caused poor data quality. Approximately 10 percent of the participants blinked during the RGB image. Blinking during the RGB image renders absolute measurements inaccurate (relative measurements could still be recorded). Also, one participant recorded the eye with the pupil partially out of frame. This participant also had the pupil partially occluded with eyelashes, but this presents two separate possible areas of failure. This problem is exemplified in image b of Figure 11, where the pupil is in frame, but not well centered and eyelashes occlude the view of the pupil.

#### 5.3.1 Smartphone Inexperience.

By far, the most prevalent user interface problem was inexperience with smartphones (N=7) that lead participants to have difficulties turning on and generally using the smartphone, even with instruction from the researcher via Zoom. Possibly the most difficult part of teaching the participants to use the smartphone was tapping (rather than firmly pressing down) and swiping on the smartphone screen.

One participant even accidentally held the volume button while turning on the phone and rebooted the phone into safe mode. With the zoom instruction, we were able to resolve this and all other issues that arose.

#### 5.3.2 Improper Testing Environment.

A few participants were unable to create a proper testing environment (N=3). Two participants had cats that continuously pestered the participants throughout their test. After being asked to avoid the cat during testing, one participant set the cat in another room, only to have the cat return and cause further issues during the test. Another participant reported loud noise outside their window, which could have affected the pupillary response.

#### 5.3.3 Scope Attachment.

A common issue during the test set up was difficulty understanding how to attach or use the smartphone scope. The scope slides over top of the smartphone, then should be placed over the eye during the test. For a few users (N=4), this was confusing even with a provided picture shown by the app. However, once the scope was attached properly, all participants were able to position the smartphone camera directly in front of their eye to record the pupil. We also found that it helps to label the phone with a red tape and a matching red tape on the attachment to provide a fiducial marker for instructional indication for where the scope should go.

#### 5.3.4 Instructions.

Some participants (N=4) misunderstood instructions within the smartphone applications. Most of the confusion revolved around when to hold the phone to the eye. Two participants held the phone to their eye while on the instruction screen, expecting a test. All participants held the phone to the eye during the testing period, but one participants initially did not hold the phone close enough to the eye (the scope must be touching the face). This was not a major issue because the smartphone had a function to check the proximity of the eye so the test would pause when the phone was too far away.

#### 5.3.5 App crashing.

During the actual testing of the digit span, a common issue participants had was the app stopping mid test. (N=3). This was likely caused by holding the lock screen button (located on the side of the smartphone) during the testing. This causes the app to go into the background and locks the phone. In all cases, the participant was able to restart the test and successfully finish.

## 6 DISCUSSION

### 6.1 Improvement from Prior Art

#### 6.1.1 Absolute Size Measurements.

Sine we are the first to create and validate a smartphone pupillometer with absolute measurements, it is difficult to directly compare to other work. Some prior works used the ground truth measurement to scale their measurement to millimeters [11]. For the PupilScreen system Mariakakis et. al. [15] convert pixels to millimeters based on a single, unvalidated constant and their ground truth is based on smartphone image used for calculating the measurement. Even so, the PupilScreen system reports a MAE during a light test of 0.62mm. Our system is validated against a gold standard pupillometer with pixel converted to millimeters based on a distance measurement and achieves a MAE of 0.39mm during a pupillarly light reflex test.

#### 6.1.2 Eye Color Agnostic.

**Figure 1:**
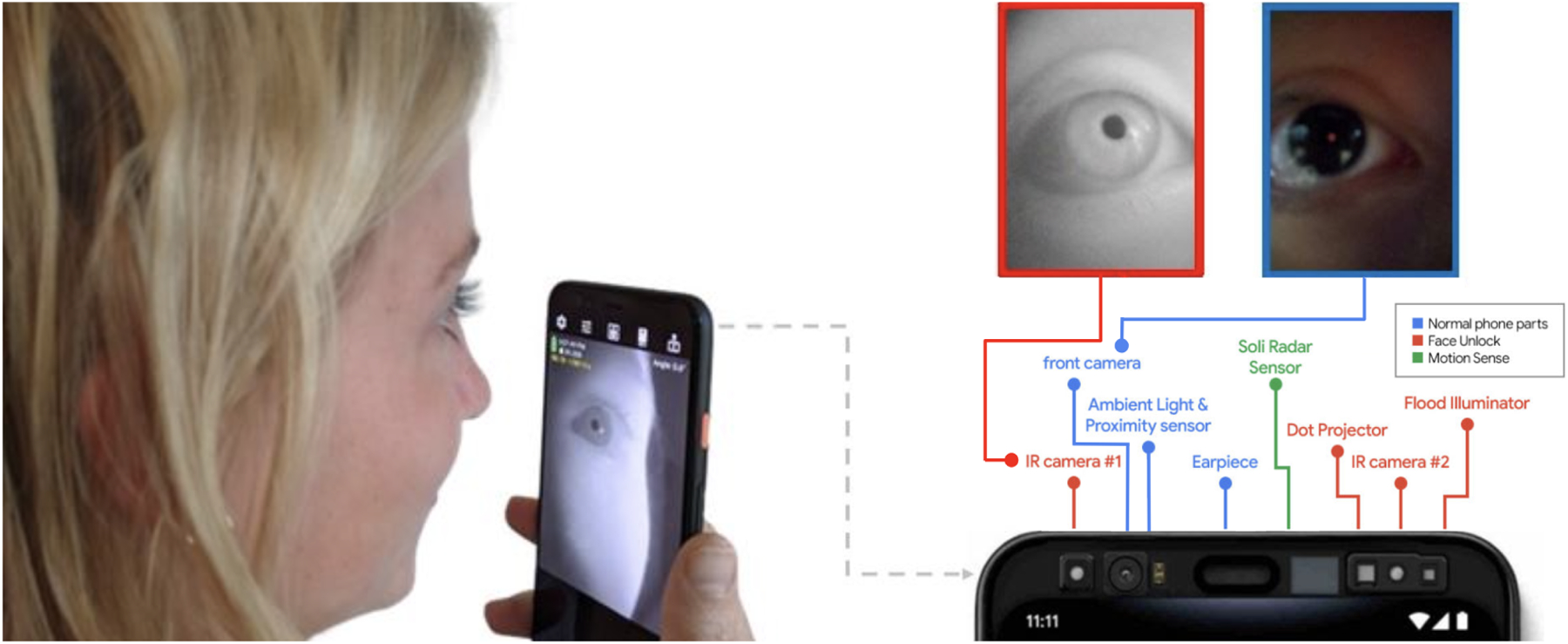
A smartphone user can image the eye using the RGB selfie camera and the front-facing NIR camera included for facial recognition. Part of this figure is adapted from [1].

**Figure 2:**
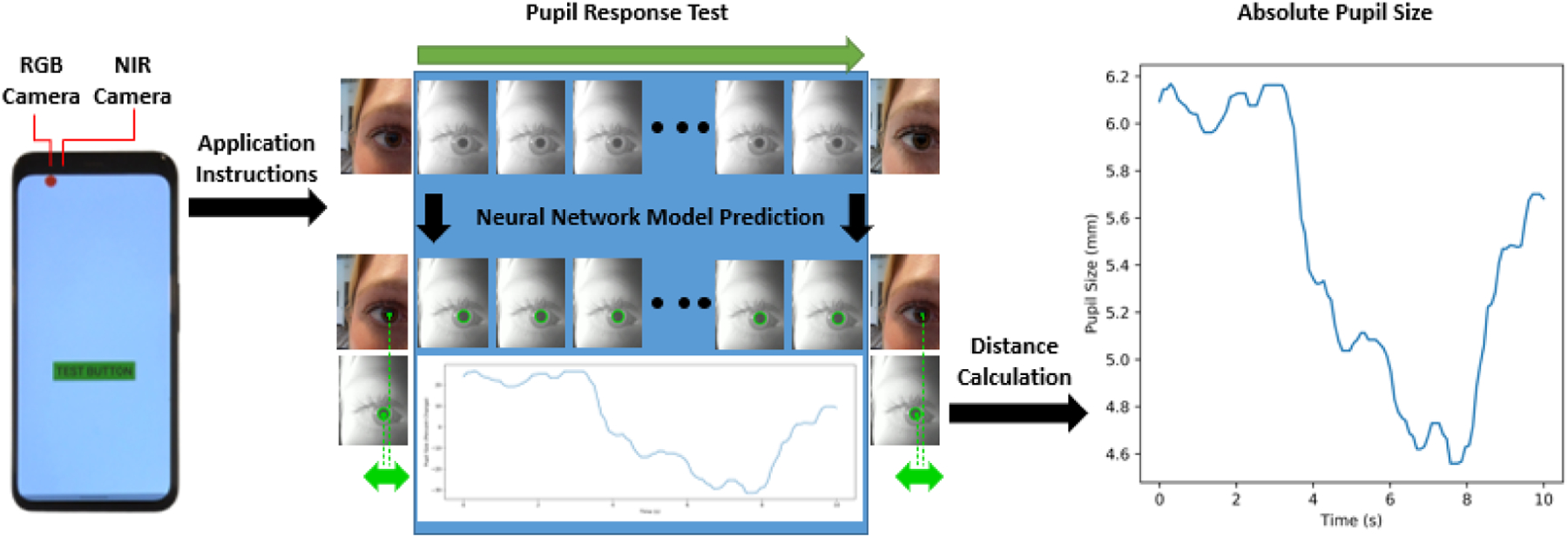
This diagram describes the flow from data acquisition to the final data. After learning receiving information and instruction from the application, users self administer a pupil response test. The data is collected of-device to compute the distance and pupil diameter. The final result is shown on the far right.

**Figure 3:**
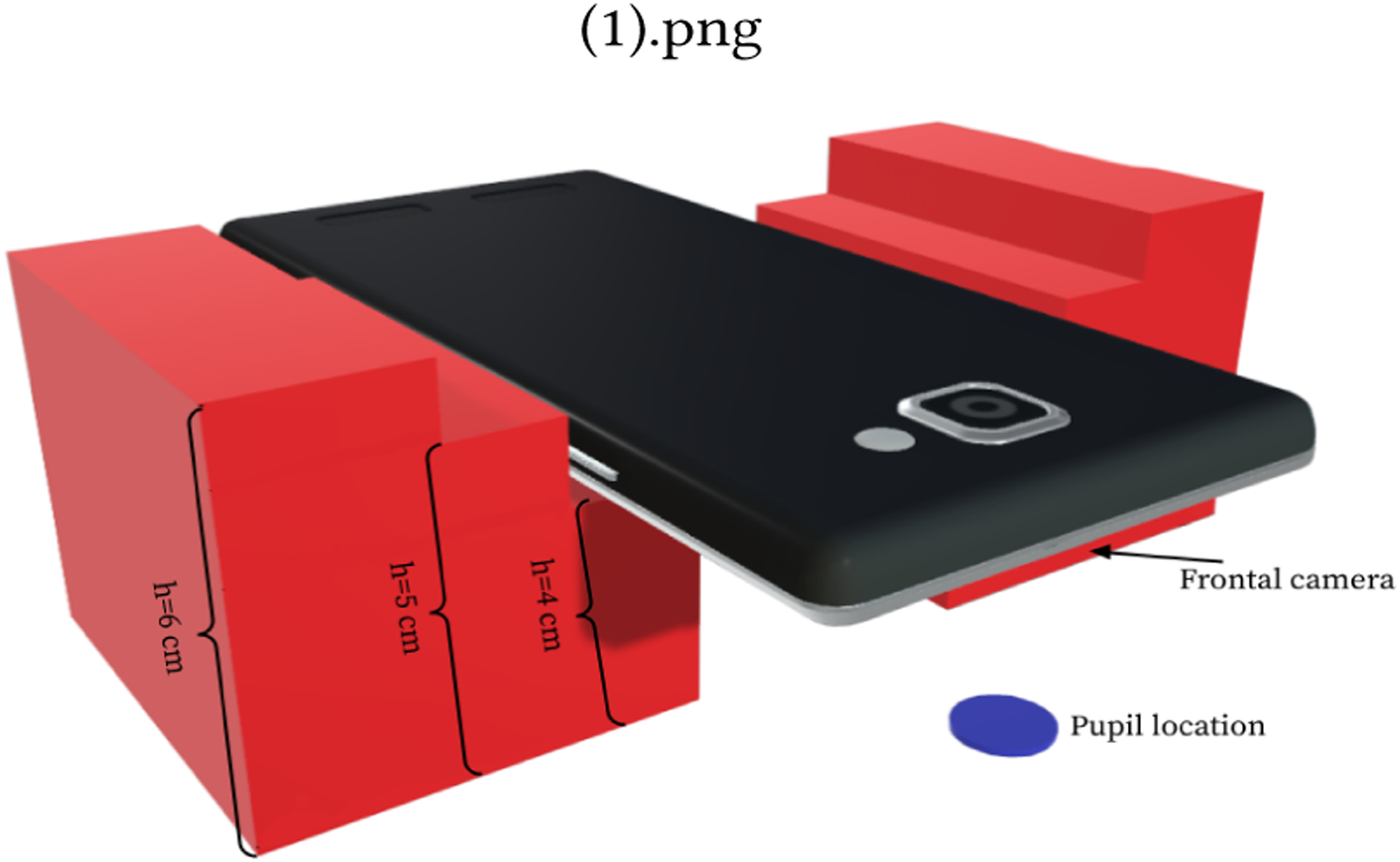
For distance validation, the phone is positioned at known distances above a printed image of a pupil.

**Figure 5:**
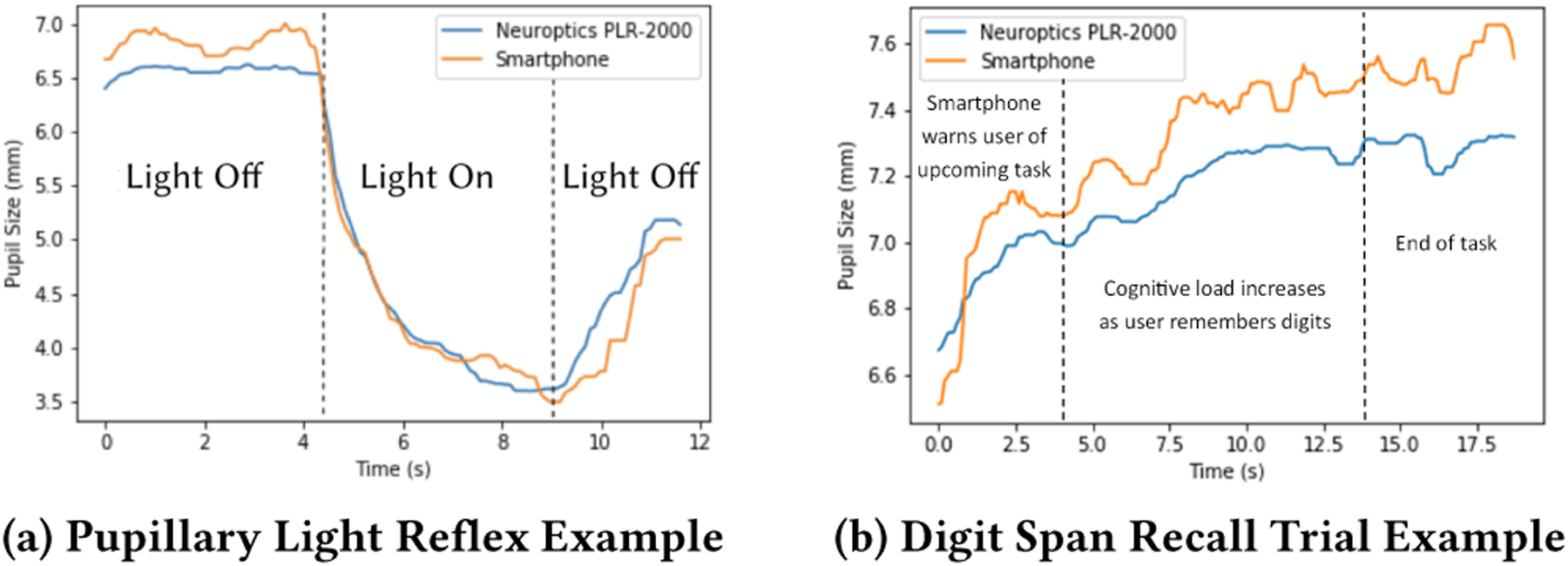
Figures (a) and (b) exemplify the expected pupil response during a pupillary light reflex test and digit span recall trial, respectively. Blue is the signal captured by a clinical pupillometer and orange is the signal captured by the smartphone system.

**Figure 7:**
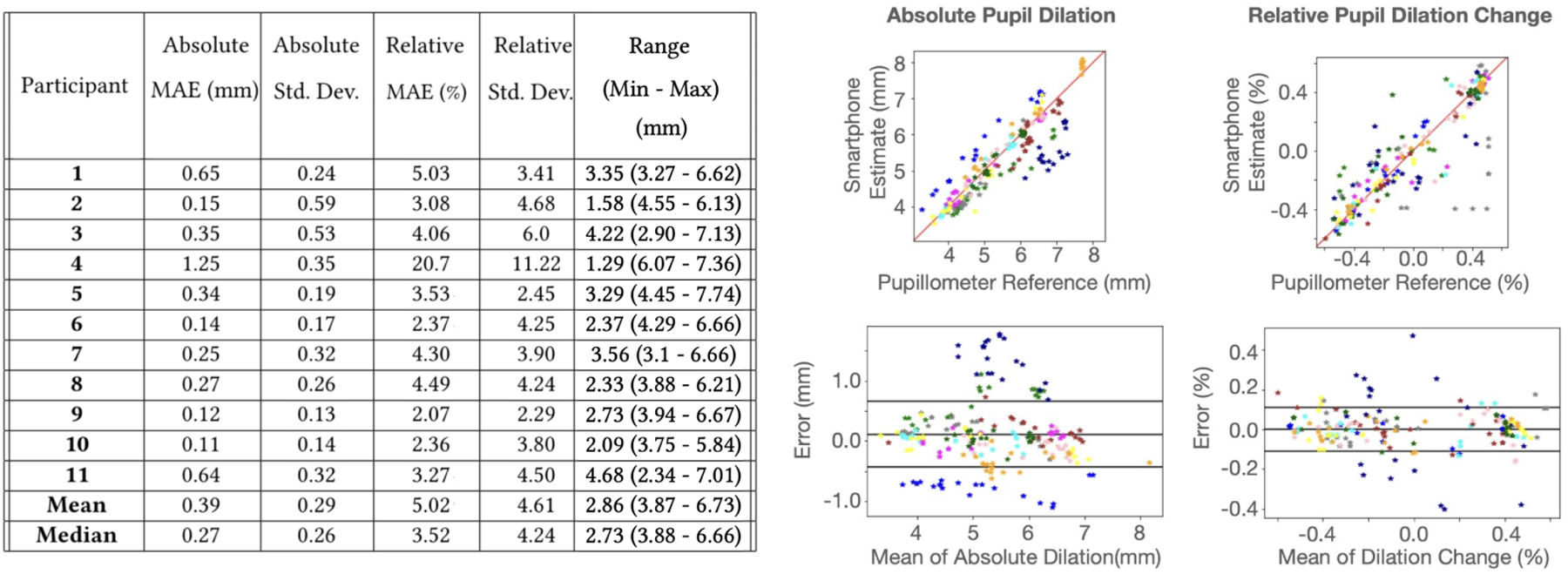
Bland-Altman and regression plots for the pupillary light reflex (PLR) tests. In all plots, different colors correspond to the different users.

Prior work on smartphone-based systems with RGB cameras, demonstrated difficulties and inaccuracies with measuring darker eye colors, some of which are excluded from analysis [19]. Using a NIR camera that allows for clear differentiation of the pupil and iris as shown in Figure 1. A Student’s T-test between light eye (N=5) and dark eye (N=6) subjects during both pupillary light reflex and digit span tests showed no statistically significant difference in both absolute and relative errors (p = 0.55, p = 0.36, p = 0.11 and p = 0.63).

#### 6.1.3 Illumination Control.

**Figure 8:**
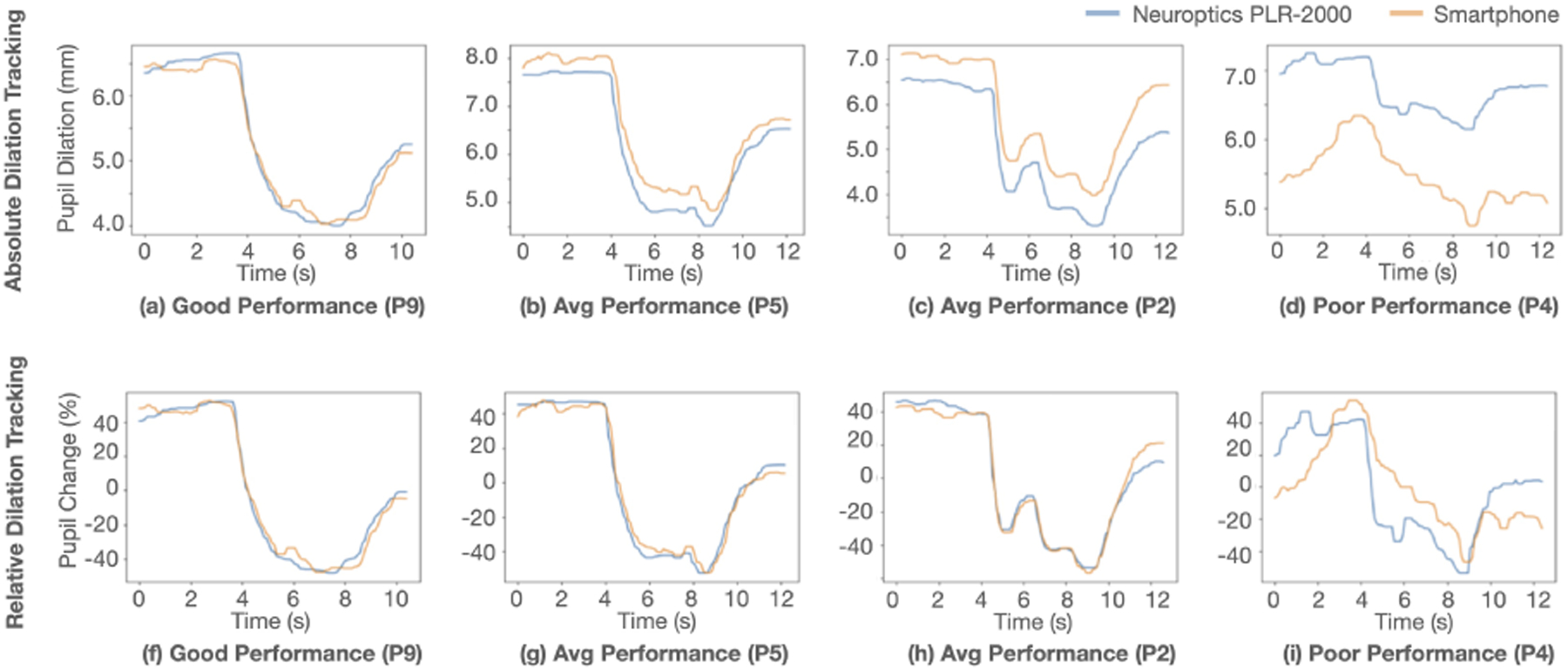
The absolute comparisons between the smartphone and the pupillometer are shown in (a), (b), (c), and (d). In (e), (f), (g), and (h), the percent change comparisons between the smartphone and pupillometer.

In all previous work we could find, the system requires the smartphone flash to be on and directed towards the user’s eye. The measurements are only recorded with the flash on. As shown in Figure 8 our system can record both high and low lighting conditions to perform a full pupillary light reflex test. The use of the NIR camera creates a measurement with less environmental requirements, which helps enable at home measurements.

### 6.2 Limitations

#### 6.2.1 Distance Measurements.

Upon viewing the data from the participants with high absolute measurement errors, it is likely that the large errors are caused by inaccurate distance estimates for certain participants. We reach this conclusion because videos showing the smartphone pupil model estimate suggest the model is accurate even when the error is high. However, the distance measurement is captured at the start of the measurement, and thus relies on the hand not moving and the eyes to look at the same spot. Because the RGB and NIR images are captured one after another, during the calibration, if the eye moves significantly between the RGB frame and the NIR frame, there will be significant error in the disparity calculation. This eye movement could be caused by hand movement, but a qualitative analysis suggests most of the error is from the participant changing where they are looking during the calibration. A small change in where the participant is looking causes a change in the center of the pupil and thus significantly changes the disparity.

**Figure 9:**
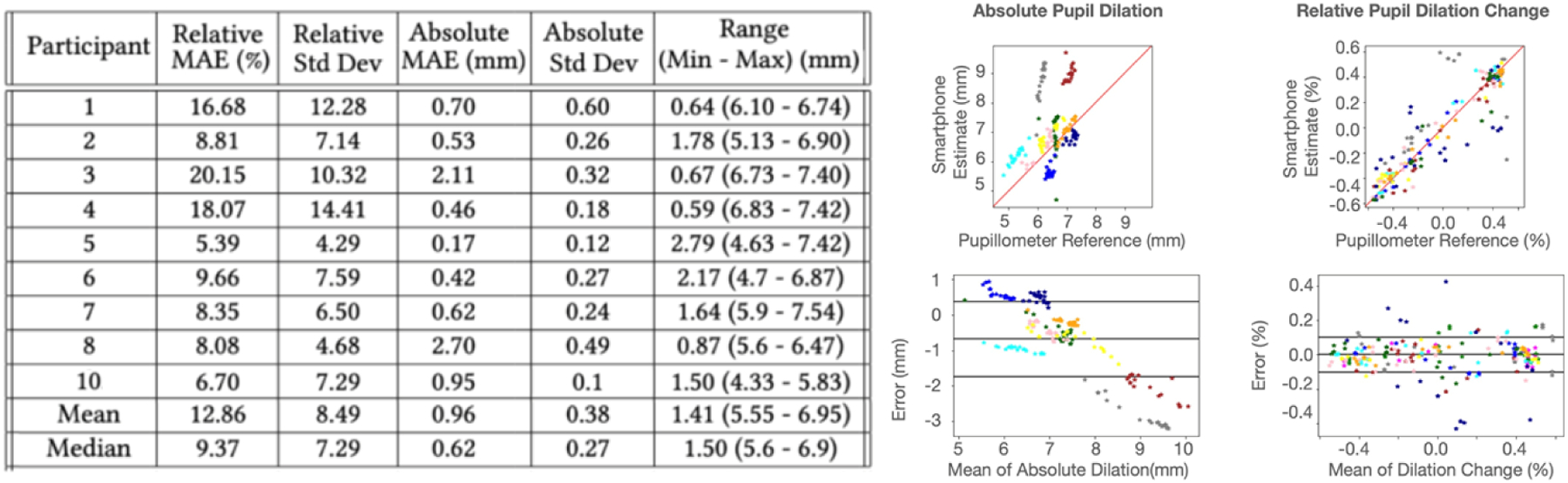
Bland-Altman and regression plots for digit span (DS) recall tests. In all plots, different colors correspond to the different users. Participants 9 and 11 are removed due to equipment failure.

**Figure 10:**
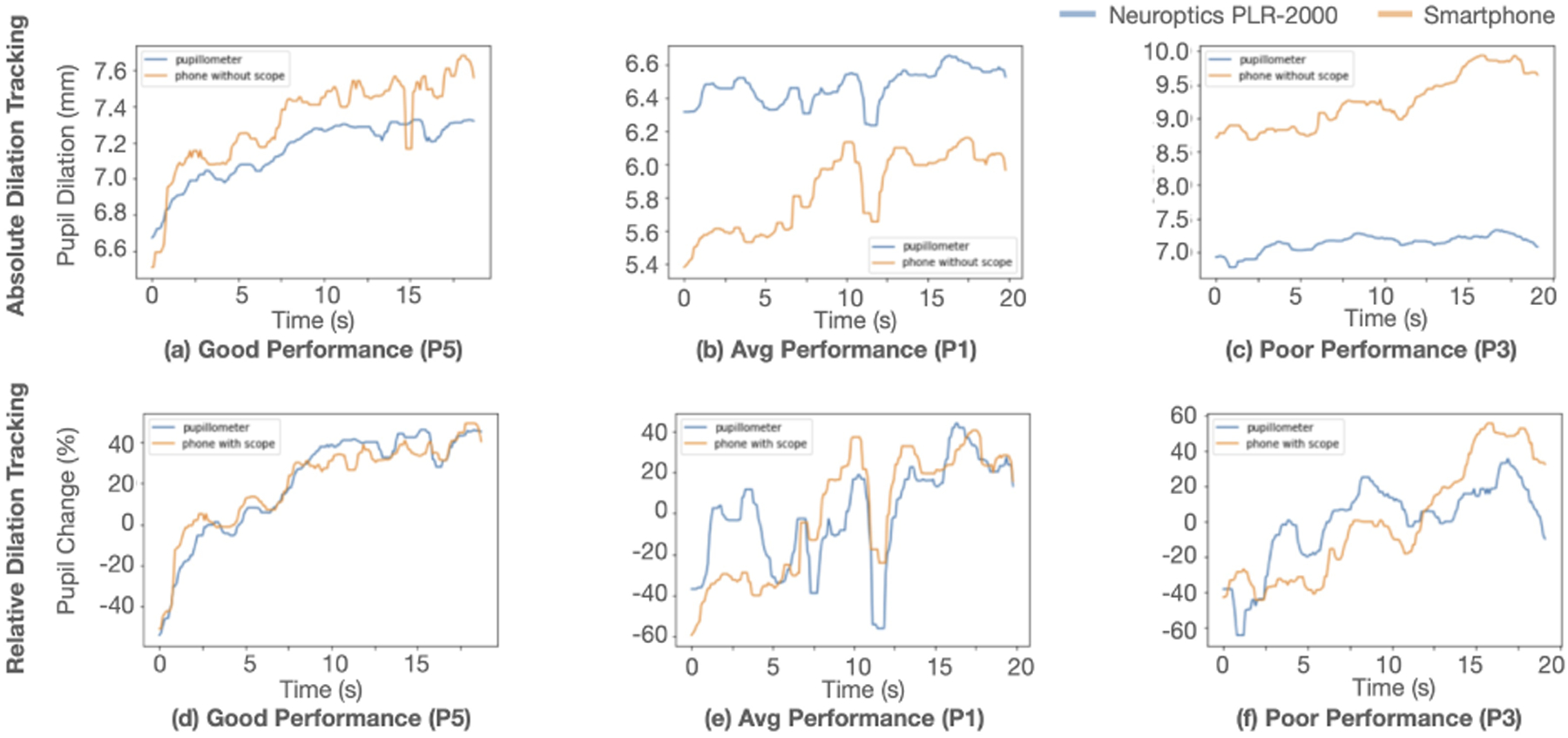
Absolute Comparisons (a–d) and relative comparisons (e–h) showing examples of good (P5), medium (P1), and poor (P3) performances. The performances are evaluated based on MAE as shown in Figure 9.

**Figure 11:**
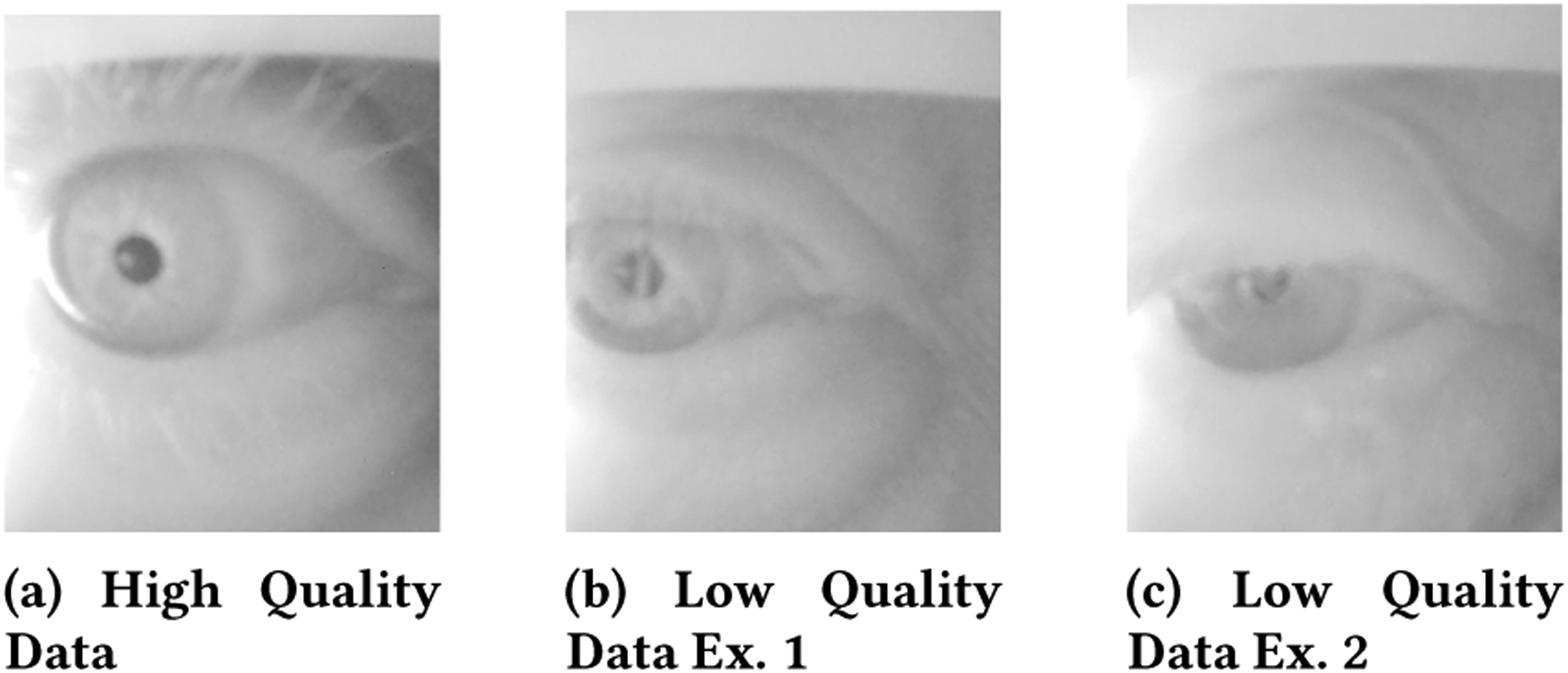
Example images from smartphone deployment data. Image a) exemplifies high quality data. b) and c) exemplify low quality data that provide lessons for how to improve for future deployments.

In addition, even with perfect calibration, since the distance is measured once at the beginning, changing the distance between the phone and the eye during the recording of the eye will cause significant error in pupil size. We suspect that this led to the higher error found in the Digit Span test (MAE = 0.96mm) compared to the Pupillary Light Reflect test (MAE = 0.27mm). As the subject focuses on listening to the digits being called out by the app, we noticed that subjects tended to move their head, pulling their eyes closer to the phone. When we look at the regression plot shown in Figure 9, we can see that there is a higher slope in a few of the subjects. This increase in trajectory is likely due to the eyes being pulled in as the test progressed leading to an overestimation of the dilation during higher cognitive load.

For future work, we plan to explore alternative methods of calculating distance including using the corner of the eye as the reference landmark or calculating pupil to iris ratio after an initial distance calibration.

#### 6.2.2 Scope Attachment.

Although our validation study is performed without any attachments, we resorted to utilizing a scope attachment during deployments to older adults. For our specific circumstance, we currently consider the scope to be an essential piece for usability purposes. Without the scope attachment, teaching a participant with limited smartphone experience with smartphones to record pupil measurements is extremely difficult. Ideally, this system would be deployed without a scope to minimize specialized equipment. Deploying without a scope may be a possibility for studies that involve younger populations with more experience with smartphones, such as for childhood ADHD studies.

#### 6.2.3 Hardware Limitations.

Despite possible improvements, there are a number of hardware limitations for our work based on the current status of smartphone-based health research. Although smartphone-based health measures offer promise for future remote health deployments that could improve healthcare with low cost, this is likely infeasible in the smartphone environment today [2]. Realistically, our application could only function on a small subset of the available smartphones on the market that have NIR cameras for facial recognition. There is also issues with commercial smartphones such that developers often do not have access to all of the hardware within the phone. For example, we were unable to access the iPhone’s facial recognition camera. In addition, even though we were able to access the NIR camera on the Google Pixel 4, we were unable to access the RGB camera to simultaneously record video from the RGB and NIR cameras for frame by frame distance calculations with stereovision.

### 6.3 Lessons from the Usability Study

The usability study demonstrates promise for future smartphone deployments to remotely collect data from older adults, but it also exposed a number of issues.

#### 6.3.1 Imaging Angle.

**Figure 12:**
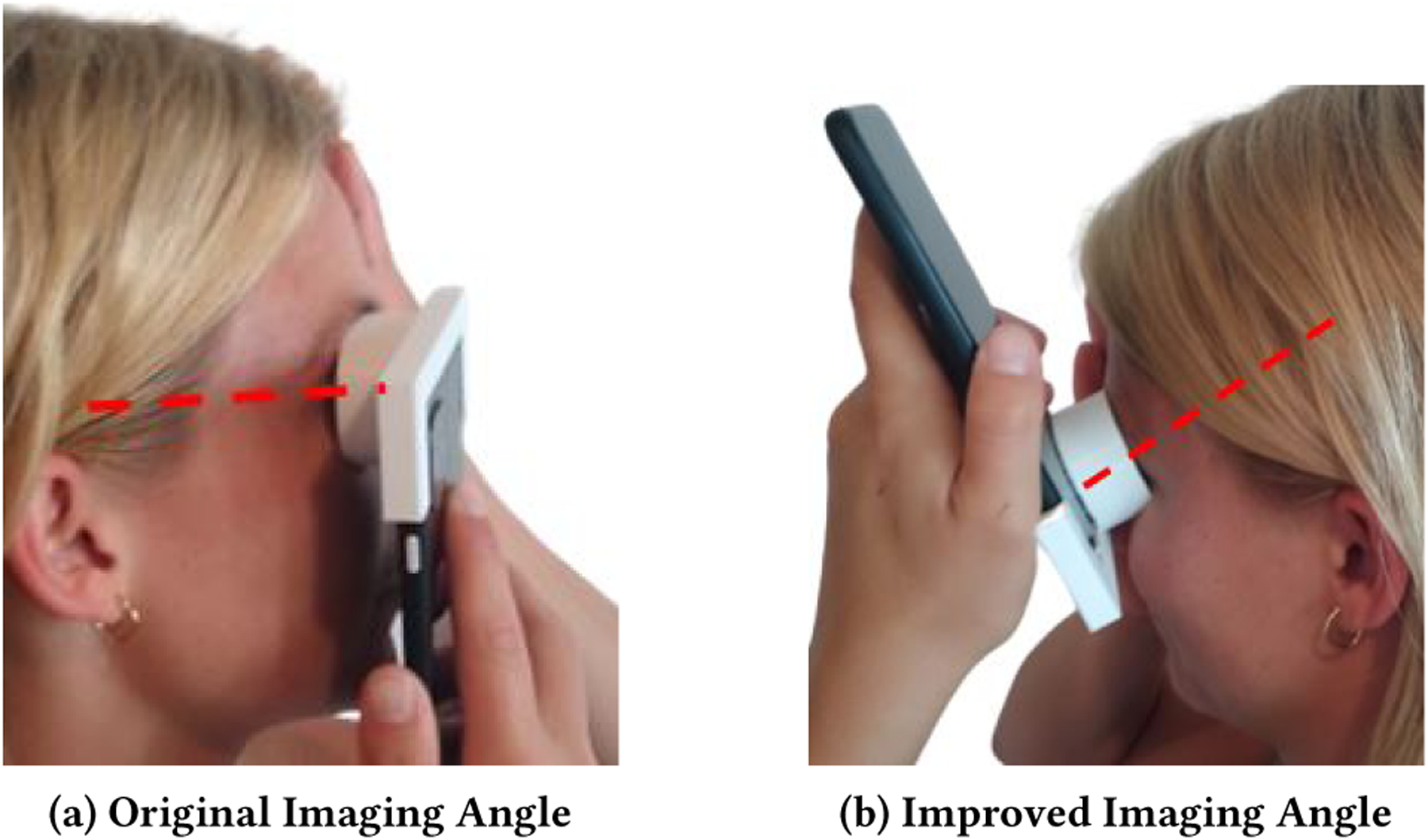
a) The original scope and phone angle that created some issues with low hanging eyelids/eyelashes b) Possible improvement to the imaging angle. The smartphone is upside down so the camera can be angled slightly upwards without hitting the face.

The most disruptive issue identified from the deployment data is the low hanging eyelid and eyelashes of older adults that hinder pupil detection and size estimation. One possible solution may be possible to solve with an amendment to the scope attachment that forces the participant to image the eye from below as shown in Figure 12. Pointing the camera up at the eye will allow for a better viewing angle of the pupil despite a low eyelid. In addition, we found that by turning the phone upside down, the phone has more clearance from the face to give a more dramatic angle to further avoid the eyelid and eyelashes.

#### 6.3.2 Blinking.

Another issue identified during the deployment was blinking. For longer tests, some blinking must be accepted, but the particular problem was blinks during the calibration when the RGB camera takes a picture. Blinking during the calibration renders the distance measurement infeasible. To avoid blinking during the critical calibration photo, the exact start of the video recording should be more clearly communicated to the participants in advance. The start of the calibration should be clearly labeled with a sound or word so participants know to keep their eyes are open at that moment.

### 6.4 Future Work

Our work showcases the potential for high accuracy smartphone pupillometry by repurposing NIR cameras in newer smartphones included for facial recognition applications, but it also emphasizes the need for improvement in a few areas.

In our current work, we explore the feasibility of smartphone pupillometry for deployments. In the future, we hope to make improvements to our system to allow for more validated deployments that would allow for research insights. For this, there are a large number of available applications. With just the pupillary light reflex test and digit span recall test, there are possibilities for investigating links to Alzheimer’s disease, ADHD, Parkinson’s Disease, schizophrenia, and more [17] [8] [31] [25] [6] [13].

## 7 CONCLUSION

In this paper, we demonstrate the feasibility of smartphone-based pupillometry. We validate the smartphone-based system through absolute and relative comparisons to a dedicated clinical pupillometer. We perform a usability study to present the challenges and possibility of deploying a smartphone-based system for research applications. The hope is that our work will inspire future exploration into mobile pupillometry research that can reach more people and investigate ideas beyond laboratory settings. Soon, mobile pupillometry could be a widespread healthcare tool available to everyone.

## Figures and Tables

**Figure 4:**
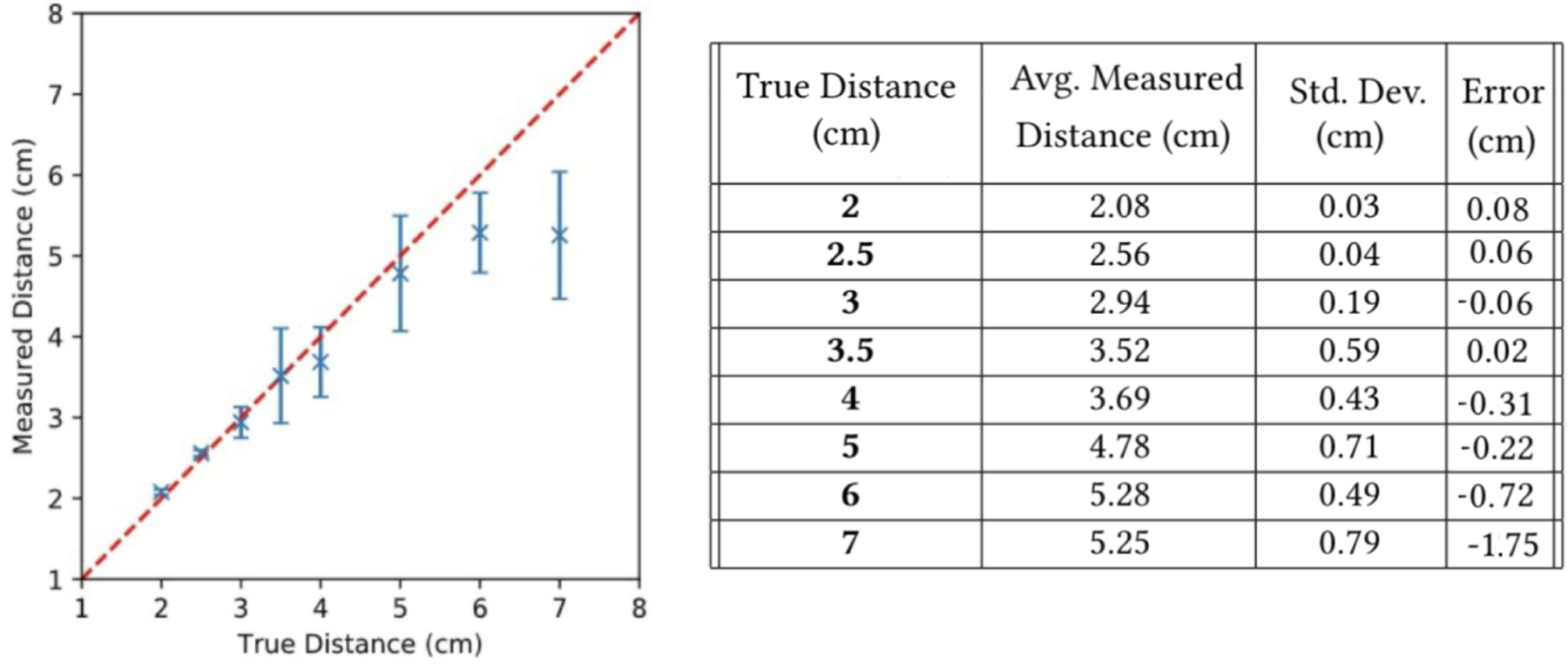
Outcome of distance measurement validation.

**Figure 6:**
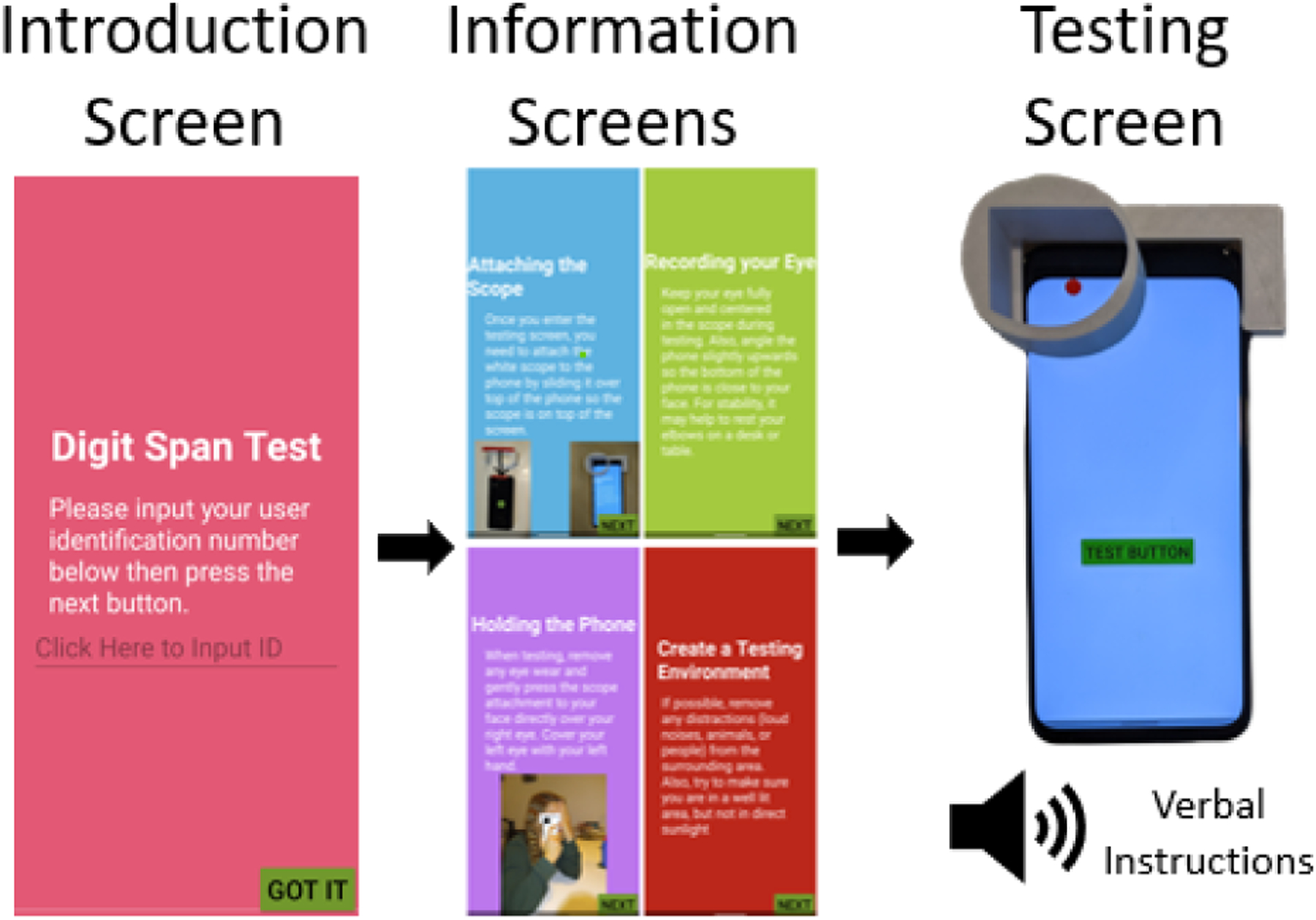
Deployment Application Overview

**Table 1:** Validation Study Participant Information

Participant	Gender	Age	Eye Color
1	Male	23	Blue
2	Female	22	Hazel
3	Male	26	Dark Brown
4	Male	23	Blue
5	Female	26	Brown
6	Male	23	Blue
7	Female	24	Brown
8	Female	23	Dark Brown
9	Male	23	Brown
10	Male	18	Dark Green
11	Male	18	Dark Brown

**Table 2:** User issues observed during the usability study

Issue	Total Participants
Difficulty unlocking or turning on the device	7
Issues During Test	3
Zoom Video Issues	2
Confusion Attaching or Using Scope	4
Misunderstanding Instructions	4
Noises or Disturbances in the Environment	2
Head Movement During Testing	2
